# 2292. Measuring Effectiveness against a Shifting Variant Landscape: COVID-19 Example in the Department of Veterans Affairs

**DOI:** 10.1093/ofid/ofad500.1914

**Published:** 2023-11-27

**Authors:** Scott L DuVall, Julie A Lynch, Patrick R Alba, Michael E Matheny, Marc A Suchard, Amanda R Shields, Aaron W C Kamauu, Lisa Glasser, Catia Ferreira, Sudhir Venkatesan, Carla Talarico, Sylvia Taylor

**Affiliations:** Department of Veterans Affairs and University of Utah School of Medicine, Salt Lake City, Utah; Department of Veterans Affairs Salt Lake City Health Care System, University of Utah School of Medicine, Salt Lake City, Utah; Department of Veterans Affairs, New York, New York; Department of Veterans Affairs, New York, New York; Department of Veterans Affairs, New York, New York; Ikaika Health, Chapel Hill, North Carolina; Ikaika Health, Chapel Hill, North Carolina; Vaccines and Immune Therapies, BioPharmaceuticals Medical, AstraZeneca, Wilmington, DE, USA, Wilmington, Delaware; AstraZeneca, Wilmington, Delaware; AstraZeenca, Cambridge, England, United Kingdom; Vaccines and Immune Therapies, BioPharmaceuticals Medical, AstraZeneca, Gaithersburg, MD, USA, Gaithersburg, Maryland; Medical Evidence, Vaccines and Immune Therapies Unit, AstraZeneca, Cambridge, UK, Cambridge, England, United Kingdom

## Abstract

**Background:**

Measuring the effectiveness of preventing and treating viruses like SARS-CoV-2 poses a challenge in understanding the variants’ susceptibility and resistance to being neutralized. Ideally, each breakthrough case would be sequenced, but in real-world settings, we rely on surveillance samples and estimated date cutoffs to determine effectiveness. This study evaluated how effect estimates would change when employing different methods for calculating variant wave dates in the same matched population.

**Methods:**

A previously published propensity score matched population1 of 2,907 immunocompromised (IC) patients exposed to 600mg of tixagevimab / cilgavimab, and 2,907 unexposed IC controls in the Department of Veterans Affairs (VA) was used for all analyses. Time to COVID hospitalization and censoring events - end of study data (Jan 2, 2023), death, or receipt of second dose - were assigned to the dominant variant circulating at the time. Traveler-based2 and National SARS-CoV-2 Strain3 genomic surveillance data from the CDC and genomic surveillance conducted in VA for the U.S. and for ten HHS regions were used. When a variant accounted for >50% of sequenced cases, all person-time during that week was assigned to that variant. Effect estimates for each variant wave were calculated using Cox proportional hazards regression models.

**Figure:**
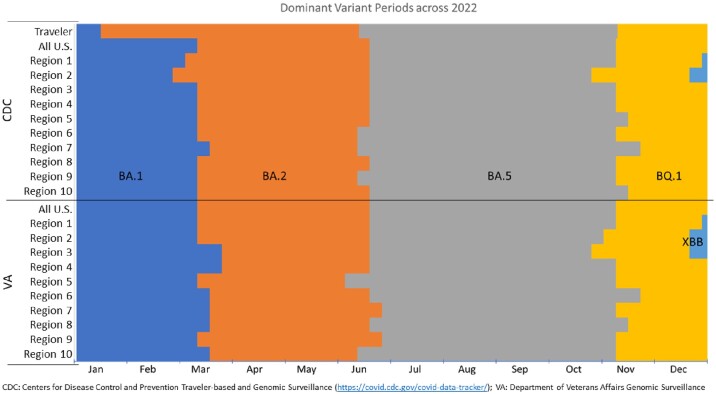

**Results:**

Dates for national and regional variant waves varied slightly, illustrating patterns of spatiotemporal spread. National CDC and VA variant dates aligned. Traveler-based surveillance detected BA.2 as the dominant strain 41 days before any other method. The start of the variant waves varied by an average of 19.25 days, while the end of the variant waves varied by 32 days on average across methods. The person count contributing time to variant waves differed by up to 669, and person-time contributing to variant waves differed by as much as 23,574 person-days. Effect estimates were tightly clustered with a maximum difference of 0.096 with similar statistical significance across variant waves and methods.

**Figure:**
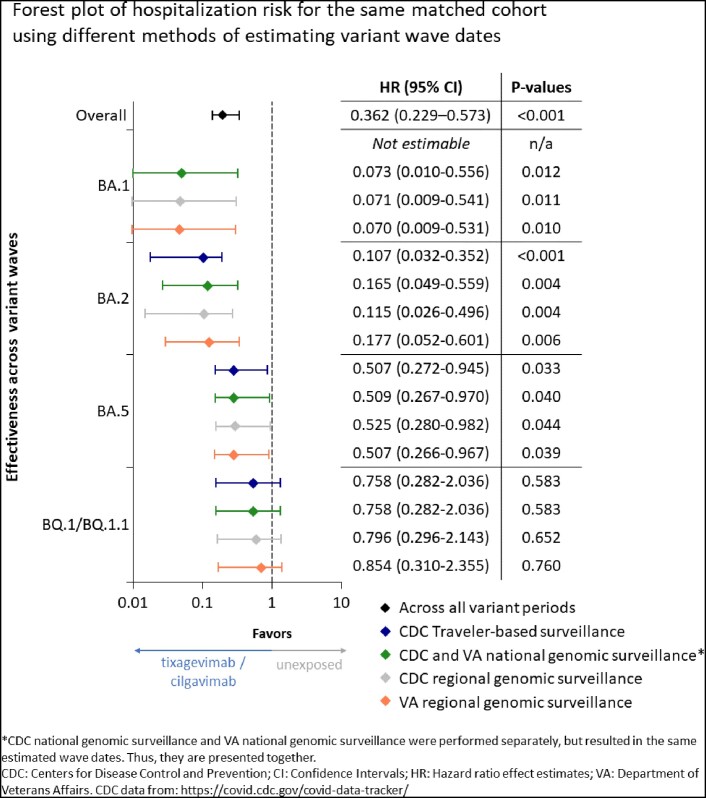

**Figure:**
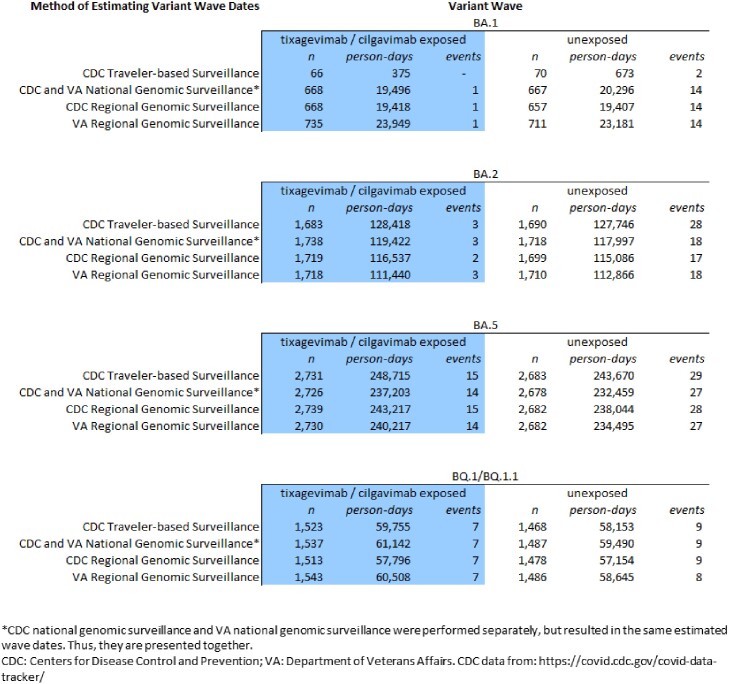

**Conclusion:**

Different methods of defining variant waves can substantially alter the number of persons and person-time contributing to each wave. However, we found that effect estimates remained robust across methods.

**Disclosures:**

**Scott L. DuVall, PhD**, Alnylam Pharmaceuticals, Inc.: Grant/Research Support|Astellas Pharma, Inc.: Grant/Research Support|AstraZeneca Pharmaceuticals LP: Grant/Research Support|Biodesix: Grant/Research Support|Celgene Corporation: Grant/Research Support|Cerner Enviza: Grant/Research Support|GSK: Grant/Research Support|Janssen Pharmaceuticals, Inc.: Grant/Research Support|Novartis International AG: Grant/Research Support|Parexel International Corporation: Grant/Research Support **Julie A. Lynch, PhD, RN, MBA; ORCID: 0000-0003-0108-2127**, Alnylam Pharmaceuticals, Inc.: Grant/Research Support|Astellas Pharma, Inc.: Grant/Research Support|AstraZeneca Pharmaceuticals LP: Grant/Research Support|Biodesix: Grant/Research Support|Celgene Corporation: Grant/Research Support|Cerner Enviza: Grant/Research Support|GSK: Grant/Research Support|Janssen Pharmaceuticals, Inc.: Grant/Research Support|Novartis International AG: Grant/Research Support|Parexel International Corporation: Grant/Research Support **Patrick R. Alba, MS**, Alnylam Pharmaceuticals, Inc.: Grant/Research Support|Astellas Pharma, Inc.: Grant/Research Support|AstraZeneca Pharmaceuticals LP: Grant/Research Support|Biodesix: Grant/Research Support|Celgene Corporation: Grant/Research Support|Cerner Enviza: Grant/Research Support|GSK: Grant/Research Support|Janssen Pharmaceuticals, Inc.: Grant/Research Support|Novartis International AG: Grant/Research Support|Parexel International Corporation: Grant/Research Support **Marc A. Suchard, MD, PhD**, Janssen Research & Development: Grant/Research Support **Amanda R. Shields, BA**, AstraZeneca: Grant/Research Support **Aaron W C Kamauu, MD, MS, MPH**, AstraZeneca: Advisor/Consultant **Lisa Glasser, MD**, AstraZeneca: Stocks/Bonds **Catia Ferreira, MSc, PhD**, AstraZeneca: Stocks/Bonds **Sudhir Venkatesan, BDS, MPH, PhD**, AstraZeneca: Stocks/Bonds **Carla Talarico, PhD, MPH**, AstraZeneca: Stocks/Bonds **Sylvia Taylor, PhD, MPH, MBA**, AstraZeneca: Stocks/Bonds

